# Patient-reported duplication and missed breast cancer survivorship care more than 5 years after initial treatment

**DOI:** 10.1007/s11764-026-02038-w

**Published:** 2026-05-11

**Authors:** Lauren P. Wallner, Allison K. C. Furgal, Christine M. Veenstra, Allison W. Kurian, Ann S. Hamilton, Kevin C. Ward, Sarah T. Hawley, Steven J. Katz, Archana Radhakrishnan

**Affiliations:** 1 Department of Internal Medicine and Rogel Cancer Center, University of Michigan, 2800 Plymouth Road, Building 16, Ann Arbor, MI 447E USA; 2 Department of Epidemiology, School of Public Health, University of Michigan, Ann Arbor, USA; 3 Department of Biostatistics, School of Public Health, University of Michigan, Ann Arbor, USA; 4 Departments of Medicine and Epidemiology and Population Health, Stanford University School of Medicine, Stanford, USA; 5 Department of Population and Public Health Sciences, Keck School of Medicine, Los Angeles, USA; 6 Department of Epidemiology, Rollins School of Public Health, Emory University, Atlanta, GA USA; 7 Department of Health Management and Policy, University of Michigan, Ann Arbor, USA; 8 Department of Health Behavior and Education, University of Michigan, Ann Arbor, USA; 9 Ann Arbor VA Center for Clinical Management Research, Ann Arbor, USA

**Keywords:** Cancer survivorship care, Coordination, Duplication, Disparities

## Abstract

**Purpose:**

Little is known about the long-term coordination of breast cancer survivorship care, including duplicated and/or missed care and whether disparities exist. We investigated patients’ perceptions of survivorship care coordination more than 5 years post-initial treatment, and examined differences by survivorship care delivery model, and patient characteristics.

**Methods:**

The iCanCare study is a longitudinal study of women diagnosed with early-stage breast cancer in 2014–2015 identified in the Los Angeles and Georgia Surveillance, Epidemiology and End Results registries. Women were surveyed during initial treatment and again 6–7 years later (2021–2022) (*N* = 1412, 60% response rate). Respondents were asked how often survivorship services were ordered twice (duplication) or key aspects of care were missed, and who led their care delivery. Associations of participant characteristics and survivorship care delivery model with duplication and missed care were estimated using multivariable logistic regression.

**Results:**

Overall, 29% of participants reported duplication, and 30% reported missed survivorship care. Latina women (vs. white) and those with 2 or more comorbidities (vs. 0) were more likely to report duplicated care (aOR: 1.71, 95% CI: 1.04–2.80, comorbidities aOR: 2.04, 1.27, 3.26). Women who reported oncologist-led care (vs. PCP-led) were more likely to report duplicated care (aOR: 2.17, 1.40–3.35) but were less likely to report missed care (aOR: 0.62, 0.42, 0.93).

**Conclusion:**

A notable proportion of long-term breast cancer survivors reported duplicated and/or missed survivorship care, with Latina women more often reporting duplicated care.

**Implications for Cancer Survivors:**

These findings highlight persistent challenges of coordinating long-term survivorship care and the need for scalable strategies and incentives to promote coordination across oncology and primary care teams.

**Supplementary Information:**

The online version contains supplementary material available at https://doi.org/10.1007/s11764-026-02038-w.

## Introduction

The number of adults surviving and living long after their initial cancer diagnoses continues to increase, with over 18 million cancer survivors in the USA alone [1]. Given the multi-modal nature of cancer treatments and increasing comorbidity burden in older adults, cancer survivors are being followed by an increasing number of clinicians post-treatment [2, 3]. As a result, the coordination of cancer survivorship care across multiple clinical specialties has become more complex [4]. Care coordination is the deliberate organization of patient care activities among multiple participants to facilitate the appropriate delivery of health care services, particularly across settings and over time [5]. For cancers with a low recurrence risk, guidelines recommend shared follow-up care between oncology and primary care clinicians when clinically indicated (e.g., protracted hormone therapy) or transitioning care to primary care after the initial post-treatment period [6]. These survivorship care delivery models aim to improve efficiency and quality but require effective coordination to ensure they improve patient outcomes. The implementation of non-oncology dominant care delivery models has lagged in the USA for lower-risk survivors [7], despite evaluation and adoption of alternative models in other countries [8–11].

A robust literature has documented gaps in the quality of survivorship care. Yet studies that specifically evaluate the coordination of survivorship care are lacking [12], and studies examining survivors’ experiences with care coordination challenges are limited. Breast cancer survivors are a particularly informative population for studying survivorship care coordination because they make up the largest group of survivors, most have favorable prognosis and thus long survival, and their care involves multiple clinical specialties over extended periods of time which increases the risk for care coordination challenges. Poor care coordination can lead to costly inefficiencies, duplication of services provided, and key aspects of survivorship care being missed, putting patients at risk of poor outcomes [4]. Two important consequences of poor care coordination, duplicated and missed care, are measurable constructs of poor care coordination but have been understudied in population-based survivorship research to date, particularly amongst longer-term survivors. Population-based studies evaluating the extent to which patient experiences of duplication and/or missed survivorship care vary across key patient clinical and sociodemographic factors are also lacking.

Therefore, we examined patient-reported care duplication and/or missed care during longer-term breast cancer survivorship in a diverse, population-based, longitudinal cohort of women with a history of early-stage breast cancer. Our goal was to examine survivors’ perceptions about and experiences with care coordination, which are not observable from medical claims or electronic medical record data alone. We also assessed variation across race/ethnicity and explored the influence of patient-reported survivorship care delivery model on patient-reported duplicated and missed survivorship care.

## Methods

### Selection and description of participants

The iCanCare study is a population-based, longitudinal study of women with early-stage breast cancer and their clinicians. As detailed previously [13–15], women ages 20–79 who were newly diagnosed with early-stage breast cancer (stages 0-II) in 2014–2015 as reported to the SEER registries of Georgia and Los Angeles County were surveyed (median time diagnosis to baseline survey completion = 7 months). African American, Asian, and Latina women were oversampled [16]. Women were ineligible if they had stage III or IV disease, had tumors larger than 5 cm, or could not complete a questionnaire in English or Spanish (*N* = 258). A total of 2502 women completed baseline surveys, resulting in a 68% response rate.

**Fig. 1:**
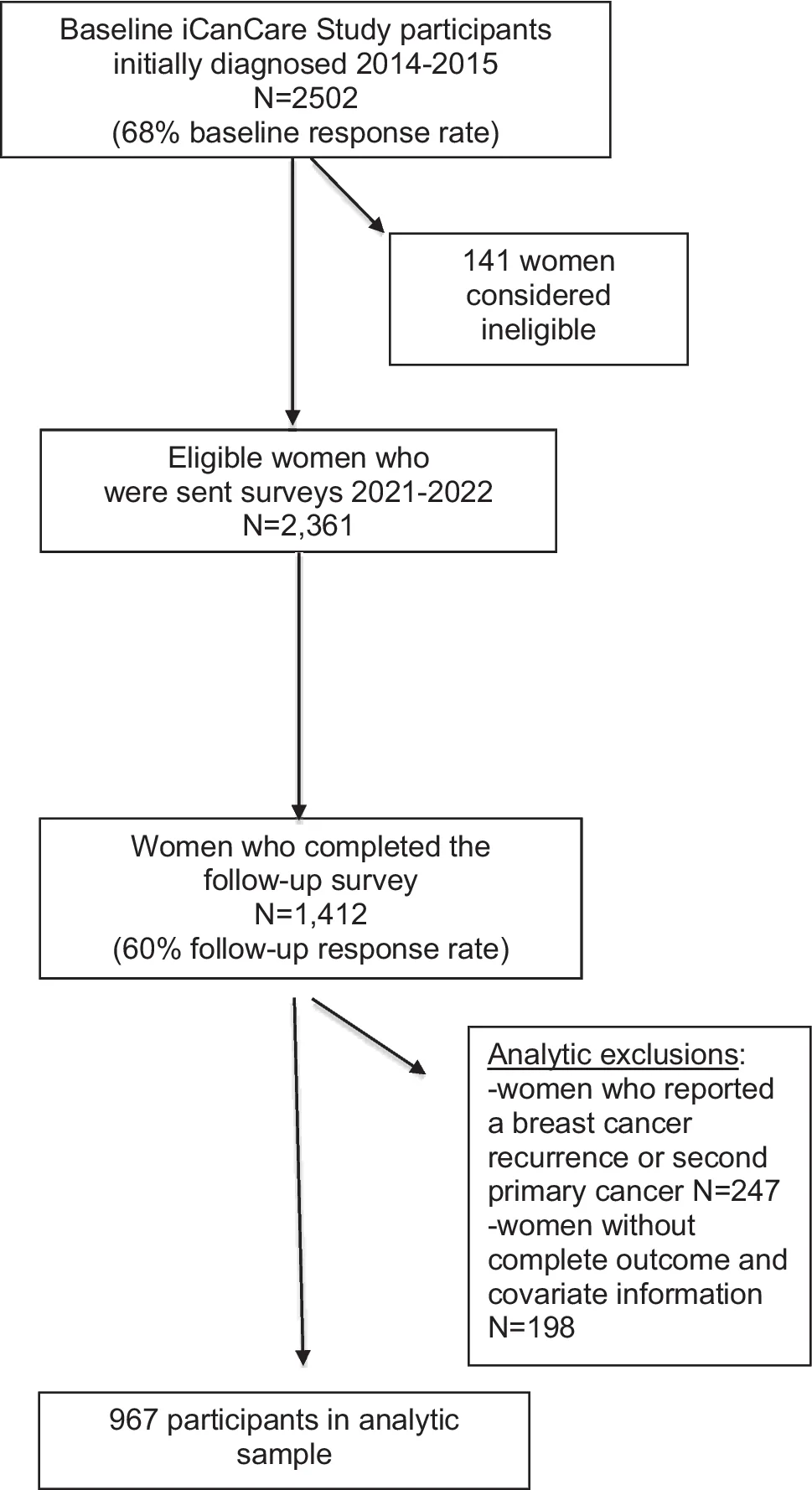
iCanCare follow-up study population

Eligible respondents were sent a follow-up survey in 2021–2022, approximately 6 years after their initial diagnosis (*N* = 2361). Women were ineligible if they were deceased (*N* = 108) or were too ill or otherwise unable to participate (*N* = 33). Eligible women were sent a packet of follow-up survey materials, with an option to complete the survey online. We again utilized a modified Dillman approach [17], including follow-up phone call reminders and survey remailings to non-respondents, and a $20 cash incentive. Of the 2361 eligible women, 1412 completed the follow-up survey, resulting in a 60% follow-up response rate (Fig. 1). For purposes of this analysis, participants who reported a breast cancer recurrence (*N* = 58), a second primary breast cancer (*N* = 29), or second primary cancer (*N* = 143) were excluded, as were those without complete outcome or covariate information (*N* = 193), resulting in 968 participants in the analytic sample. Responses to the surveys were merged with SEER clinical data, and a de-identified analytic dataset was created. The study was approved by the University of Michigan Institutional Review Board and the state and institutional IRBs of the SEER registries.

### Data collection and measurements

Questionnaire content was developed based on a conceptual framework, informed by the NCI Quality of Survivorship Care Framework [18], prior literature [9, 12, 19], and our prior work [2, 13, 20–22]. Specific domains from the NCI Quality of Survivorship Care Framework that were used to guide measure development included individual factors, healthcare delivery, including care coordination, clinical structure, and patient and caregiver experiences sub-domains. Items were designed to capture survivors’ experiences with potentially duplicative testing and missed or delayed care across providers during survivorship follow-up. Items were iteratively reviewed by investigators with expertise in cancer survivorship, survey methodology, health services research, and behavioral science to ensure clarity and content validity. We then rigorously pilot tested the measures, including cognitive pretesting with 19 breast cancer survivors and a pilot study in a sample of 67 baseline participants.

Patient-reported duplication in survivorship care: Respondents were asked, “In the past two years, how often has your PCP or oncology provider ordered a medical test or procedure that the other one had already ordered?” with response options on a 5 pt. Likert type scale, ranging from never to very often. Responses were dichotomized into never vs. any duplication for analyses.

Patient-reported missed survivorship care: Respondents were asked, “In the past two years, how often do you think an important part of your breast cancer follow-up care was missed?”, with response options ranging from never to very often on a 5-point Likert-type scale. Responses were dichotomized into never vs. any missed care for analyses.

Patient-reported survivorship care delivery model: Respondents were asked, “Since you finished your primary treatment for breast cancer, which provider has been most responsible for providing your follow-up care?” Response categories included “almost always my primary care provider,” “usually my primary care provider,” “both providers have been equally responsible,” “usually my oncology provider,” and “almost always my oncology provider.” Responses were categorized as PCP-led (usually/almost always my PCP), Oncologist-Led (usually/almost always my oncology provider), and Shared (both) for analyses [2].

Patient-reported race/ethnicity: Patient-reported race/ethnicity at baseline (white, Black, Asian, Latina, other/unknown/missing) was examined as an independent variable.

Covariates: Covariates at baseline include SEER-collected factors (age at diagnosis, stage at diagnosis) and patient-reported sociodemographic factors (education, income, insurance, surgery, radiation, chemotherapy, hormone therapy). Covariates assessed at follow-up included patient-reported insurance and patient-reported number of comorbidities (0, 1, 2+). Patient-reported receipt of non-recommended testing was examined as it is an indicator of low-value care that may also reflect poor coordination. Participants were asked how long it had been since they received blood tests for signs of cancer (CA-125 or CEA test) or other imaging tests to check for breast cancer coming back (CT scan, PET scan, or bone scan), with response options of never, within the past 2 years, 2–5 years ago, more than 5 years ago [22]. The responses were dichotomized into never/more than 5 years ago vs. within the past 2 years/2–5 years ago for analyses. The impact of COVID19 on care was characterized by asking participants whether their ability to get general preventive care, breast cancer follow-up care, fill/refill medications, and communicate with primary care and oncology providers was worse or better during the COVID19 pandemic, with response options of a lot worse to a lot better (5-point Likert-type scales). A summary Impact of COVID19 on Care scale was then created using the averages and dichotomized into high (> 3) vs. low (≤ 3) impact.

### Statistical analyses

We first assessed differences in the bivariate distributions of duplication and missed care across patient clinical and sociodemographic factors using chi-square and two-sided *t*-tests as appropriate. Multivariable-adjusted logistic regression models (separate models for duplication and missed care) were then used to evaluate the associations of participant characteristics and survivorship care delivery model with duplication and missed care, adjusting for age at diagnosis, education, income, race/ethnicity, insurance, number of comorbidities, stage at diagnosis, surgery, radiation, chemotherapy, hormone therapy, and study site. We assessed the impact of COVID19 pandemic on our results by adjusting for patient-reported impact of COVID19 in sensitivity analyses. The results were not meaningfully different. Because patient-reported receipt of non-recommended testing is likely on the causal path, we did not include it in our multivariable models and descriptively assessed differences in the distributions of perceptions of duplication and missed care by patient-reported receipt of non-recommended services.

To account for possible bias related to differential response, all multivariable statistical analyses incorporated weights to account for differential probabilities of sample selection and nonresponse and to assure that the distributions of our sample resemble those of the target population. To account for the potential impact of missing data, values for missing items were imputed using sequential regression multiple imputation with IVEware in SAS [23]. We generated ten independently imputed complete datasets which were analyzed separately. Results were combined and inferential statistics calculated using PROC MIANALYZE in SAS. All analyses were performed using SAS version 9.4 (SAS Institute, Cary, NC).

## Results

Overall, 28.0% of participants reported perceiving that some aspect of their survivorship care was duplicated across primary care and oncology. Perceptions of duplicated survivorship care differed significantly by race/ethnicity and were greatest amongst Black (31.2%) and Latina participants (43.6%), compared to white participants (23.7%) (*p* < 0.001). A greater proportion of Spanish-speaking Latinas reported duplicated care compared to English-speaking Latinas (47.7 vs. 34.4%, *p* < 0.001, data not shown). The proportion of women reporting duplicated care increased with increasing number of comorbidities; 32.9% of those who reported 2 or more comorbidities reported duplicated care, compared to 20.8% of those with no comorbidities (*p* < 0.01) (Table 1).

**Table 1:** Patient perceptions of duplicated and missed survivorship care by patient characteristics (*N* = 968)

	No patient-reported duplicated care (N=696)		Patient-reported duplicated care (N=271)		No patient-reported missed care (N=678)		Patient-reported missed care (N=289)	
	N	Weighted %	N (%)	Weighted %	N (%)	Weighted %	N (%)	Weighted %
Age at diagnosis								
<40	18	73.2	7	26.8	16	67.4	9	32.6
40-49	92	69.1	40	30.9	81	61.8	51	38.2
50-59	176	67.1	81	32.9	160	62.3	97	37.7
60-69	270	73.9	89	26.1	265	73.3	94	26.7
70+	140	74.5	54	25.5	156	80.0	38	20.0
* p-value*				*0.38*				*0.001*
Race/ethnicity								
White	402	76.3	125	23.7	388	73.6	139	26.4
Black	121	68.8	52	31.2	116	67.1	57	32.9
Latina	94	56.4	66	43.6	98	61.7	62	38.3
Asian	69	73.5	23	26.5	66	69.2	26	30.8
Other/unknown/missing	10	71.0	5	29.0	10	69.5	5	30.5
* p-value*				*<0.001*				*0.09*
Education								
<= Some high school	37	58.0	29	42.0	38	58.9	28	41.1
High school graduate	124	70.4	49	29.6	136	77.2	37	22.8
Some College	195	72.5	77	27.5	193	71.9	79	28.1
College graduate	203	74.3	67	25.7	188	69.8	82	30.2
Graduate degree or higher	137	72.8	49	27.2	123	64.3	63	35.7
* p-value*				*0.15*				*0.04*
Income at follow-up								
<20K	67	62.9	41	37.1	71	64.5	37	35.5
20-40K	87	73.0	36	27.0	93	79.2	30	20.8
40-60K	115	73.4	42	26.6	116	75.1	41	24.9
60-90K	122	74.6	42	25.4	105	63.2	59	36.8
90K+	212	72.9	69	27.1	199	70.5	82	29.5
Don’t know/not reported	93	70.2	41	29.8	94	69.0	40	31.0
* p-value*				*0.39*				*0.05*
Insurance at follow-up								
No insurance	11	71.4	4	28.6	11	68.2	4	31.8
Government (Medicare,	368	71.5	147	28.5	382	73.4	133	26.6
Medicaid, VA)								33.9
Private/other	317	71.7	120	28.3	285	66.1	152	
* p-value*				*0.99*				*0.12*
Number of comorbidities at follow-up								
0	145	79.2	43	20.8	123	64.9	65	35.1
1 2+	174377	76.167.1	52176	23.932.9	172383	76.969.3	54170	23.130.7
* p-value*				*0.003*				*0.04*
Stage at diagnosis								
0	132	74.0	46	26.0	121	68.7	57	31.3
I	397	70.9	154	29.1	399	71.6	152	28.4
II	167	70.2	71	29.8	158	68.2	80	31.8
* p-value*				*0.65*				*0.62*
Surgery								
Lumpectomy	450	72.8	164	27.2	441	71.3	173	28.7
Unilateral mastectomy	109	65.2	54	34.8	116	72.0	47	28.0
Bilateral mastectomy	137	72.9	53	27.1	121	64.6	69	35.4
* p-value*				*0.18*				*0.21*
Radiation								
No	260	72.4	99	27.6	247	69.3	112	30.7
Yes	436	71.1	172	28.9	431	70.6	177	29.4
* p-value*				*0.70*				*0.69*
Chemotherapy								
No	501	73.6	172	66.1	488	72.0	185	28.0
Yes	195	26.4	99	33.9	190	65.0	104	35.0
* p-value*				*0.03*				*0.04*
Hormone therapy								
No	184	76.4	58	23.6	158	66.4	84	33.6
Yes	512	69.7	213	30.3	520	71.5	205	28.5
* p-value*				*0.06*				*0.16*
Geographic location								
Georgia	405	75.7	131	24.3	388	71.8	148	28.2
Los Angeles	291	66.4	140	33.6	290	68.0	141	32.0
* p-value*				*0.003*				*0.23*
Impact of COVID19 on care								
Low impact	424	71.9	160	28.1	452	77.0	132	23.0
High impact	259	71.0	107	29.0	212	58.3	154)	41.7
* p-value*				*0.78*				*<0.001*

Overall, 29.9% of participants reported an important aspect of their survivorship care was missed. Missed survivorship care significantly differed by race/ethnicity (*p* = 0.04) and was greatest amongst Latina (38.3%), Black (32.9%), and Asian (30.8%) women followed by white women (26.4%). A greater proportion of Spanish-speaking Latinas reported missed care compared to English-speaking Latinas (42.6% vs. 36.4%, data not shown). More women who received chemotherapy reported experiencing missed care compared to those who did not receive chemotherapy (35.0% vs. 28.0%) (*p* = 0.01) (Table 1).

**Fig. 2:**
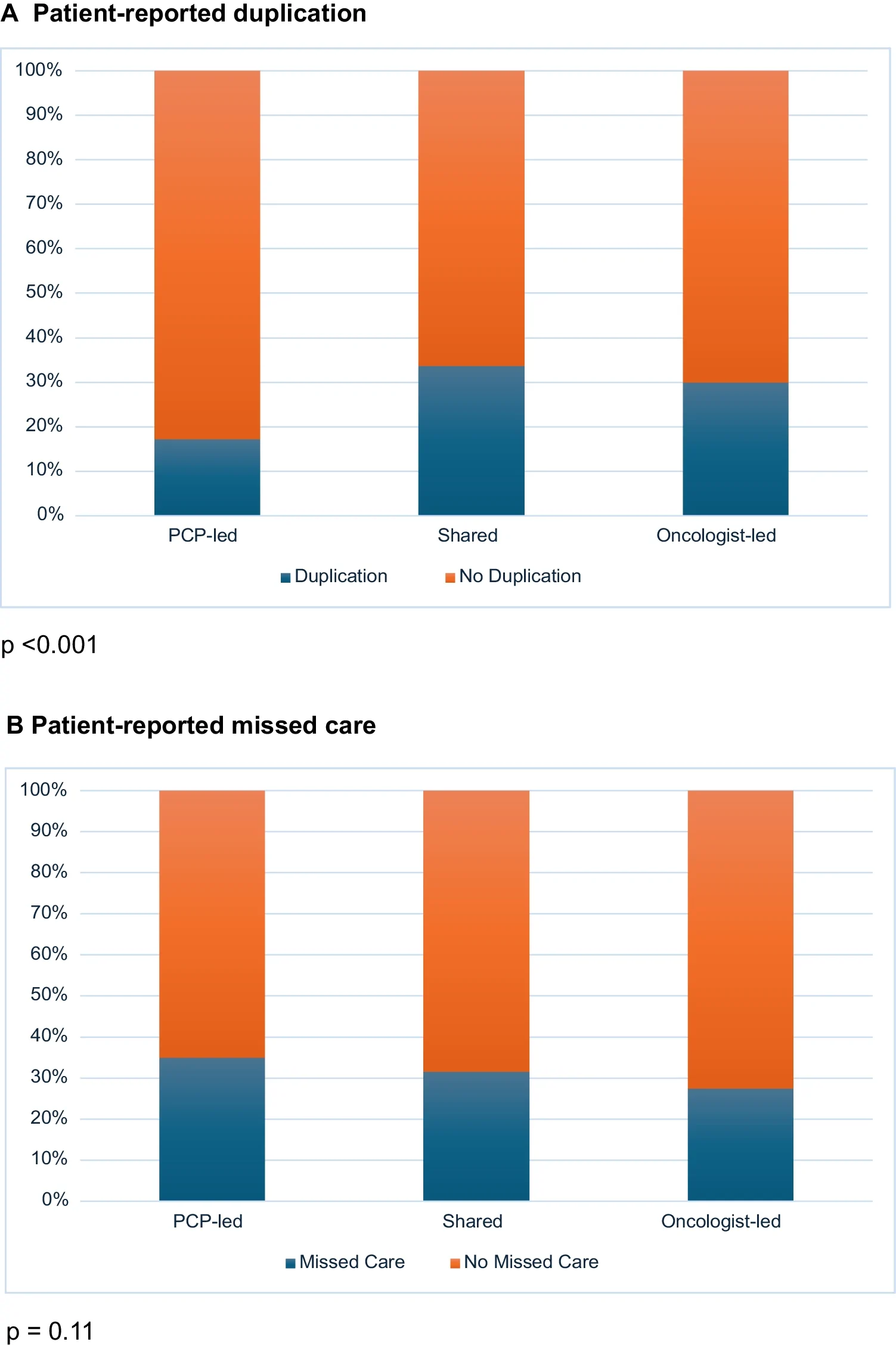
Distribution (%) of patient perceptions of duplicated (**A**) and missed (**B**) survivorship care by patient-reported survivorship care delivery model. **A** Patient-reported duplication. Weighted *p* < 0.001. **B** Patient-reported missed care. Weighted *p* = 0.25

Duplication was greatest amongst those who reported care was shared equally between primary care and oncology (34.5%), followed by those who reported oncologist-led survivorship care (30.7%), and finally by those who reported PCP-led care (16.7%) (weighted *p*-value < 0.001, Fig. 2A). Participant-reported missed survivorship care did not statistically significantly differ across patient-reported survivorship care delivery model (weighted *p* = 0.25, Fig. 2B). The proportion of participants who reported duplicated survivorship care was greater among those who also reported they received tumor marker testing in the past 5 years (36.0%) vs. those who did not (22.4%) and those who received imaging for recurrence in the past 5 years (36.2%) vs. those who did not (24.5) (both weighted *p*-values < 0.001, Supplemental Table 1). Participant-reported missed survivorship care did not significantly differ across patient-reported receipt of tumor marker testing or imaging for recurrence (Supplemental Table 1).

The odds of participants reporting duplicated survivorship care were 1.7 times greater amongst Latina compared to white participants (adjusted OR: 1.71, 95% CI: 1.04–2.80, Table 2). Survivors with 2 or more comorbidities had double the odds of reporting duplicated care compared to those who did not have any comorbidities (adjusted OR: 2.04, 95% CI: 1.27–3.26). Survivors who reported oncologists led their survivorship care delivery, or their care was shared equally between oncologists and PCPs had more than double the odds of reporting duplicated survivorship care (oncologist-led adjusted OR: 2.17, 95% CI: 1.40–3.35, shared adjusted OR: 2.61, 95% CI: 1.58–4.29). Women who reported oncologist-led survivorship care had lower odds of reporting their survivorship care was missed when compared to those who reported PCP-led care (adjusted OR: 0.62, 95% CI: 0.42–0.93) (Table 2).

**Table 2:** Pooled estimates from weighted multivariable logistic regression models of patient perceptions of duplicated and missed care using multiply imputed data adjusting for sociodemographic and clinical factors, 10 imputed datasets with average *N* = 1185

	Patient-reported duplication*	Patient-reported missed care**
	Adjusted OR (95% CI)	Adjusted OR (95% CI)
Age at diagnosis	0.98 (0.96–1.00)	0.98 (0.96–1.00)
Race/ethnicity		
White	1.0 (ref)	1.0 (ref)
Black	1.50 (0.98–2.30)	1.16 (0.74–1.80)
Latina	1.71 (1.04–2.80)	1.60 (0.97–2.63)
Asian/PI	0.93 (0.52–1.68)	1.33 (0.76–2.32)
Unknown/missing***	1.83 (0.69–4.85)	2.10 (0.76–5.81)
Number of comorbidities		
0	1.0 (ref)	1.0 (ref)
1	1.26 (0.76–2.09)	0.68 (0.42–1.10)
2+	2.04 (1.27–3.26)	1.05 (0.69–1.59)
Provider-led Survivorship care		
PCP-led	1.0 (ref)	1.0 (ref)
Oncologist-led	2.17 (1.40–3.35)	0.62 (0.42–0.93)
Shared	2.61 (1.58–4.29)	0.80 (0.50–1.28)

^*^Duplication model also adjusted for: education, income, insurance, stage at diagnosis, surgery, radiation, chemotherapy, hormone therapy, and site

^**^Missed care model adjusted for: education, income, insurance, stage at diagnosis, surgery, radiation, chemotherapy, hormone therapy, impact of COVID19 on care, and site

^***^These missing values were not imputed

## Discussion

In this population-based sample of breast cancer survivors who are more than 5 years out from initial treatment, a notable proportion perceived that some aspect of their survivorship care was either duplicated and/or missed. Perceptions of duplicated care were greatest amongst Latina survivors and those with two or more comorbidities. Survivors who reported their care was led by an oncologist were more likely to perceive duplication in their survivorship care but less likely to perceive any aspects of their care were missed when compared to those whose survivorship care was led by primary care.

Our results highlight that there are vulnerable subgroups of survivors who may be at increased risk of experiencing poor care coordination many years out from initial treatment. To our knowledge, this is the first study to find that Latina breast cancer survivors are more likely to perceive duplication in survivorship care when compared to white breast cancer survivors. While we do not know whether Latinas in this cohort actually experienced duplicated survivorship care, prior studies have demonstrated key gaps in quality across the continuum of cancer amongst Latinas, including in the receipt of cancer treatments [24, 25], high-quality treatment decision making [26, 27], and side-effect experiences [28, 29]. Latinas may remain at risk for experiencing quality gaps well into survivorship. Future confirmatory studies evaluating care duplication in diverse survivor populations using medical claims or electronic medical record data are warranted. Recent national data sources, including the 2024 Health Information National Trends Survey (HINTS) [30], now include patient-reported measures of duplication, creating new opportunities to examine these outcomes at scale. Efforts to improve care coordination for Latina breast cancer survivors specifically may also be warranted. Our descriptive results suggest Latinas who speak Spanish may more often perceive their survivorship care is duplicated. Therefore, efforts to improve care coordination need to be available in the language spoken by Latina survivors, culturally sensitive, and patient-centered to ensure the survivorship care needs of Latinas are being sufficiently addressed, and they are receiving guideline-recommended care.

To date, very few studies have measured missed survivorship care specifically. In this cohort, a third of participants reported the perception that some aspect of their survivorship care was missed. There was no variation in perceptions of missed survivorship care observed across patient characteristics, which is reassuring. The time period in this study coincides with a time of transition for many lower-risk survivors when they may transition from being primarily followed by oncologists to being followed by primary care. Survivors are likely more at risk of missed care during this time period of survivorship due to gaps in communication and coordination that often occur during care transitions. Skolarus and colleagues found that prostate cancer survivors with more fragmented care were more likely to receive duplicated surveillance testing and had increased costs compared to those receiving more coordinated care [31]. This underscores the risks associated with poorly coordinated survivorship care, which may include more morbidity and gaps in the receipt of high-quality survivorship services.

Prior research has primarily focused on characterizing utilization patterns during survivorship and their association with receipt of guideline-recommended care, and almost exclusively focuses on the first 5 years after diagnosis [32]. Our findings suggest that for breast cancer survivors who were more than 5 years out from their initial treatment, the clinician who primarily delivers their survivorship care influences whether they perceive their survivorship care is duplicated and/or missed. Oncologist-led survivorship care and shared survivorship care were associated with more duplication when compared to PCP-led care, underscoring the challenges of coordinating survivorship care across multiple clinical specialties. These findings build upon prior work, including our own, highlighting that a lack of clarity around provider roles and communication challenges are common in survivorship care delivery and lead to poorer quality of care [13, 21, 33]. Our findings that perceptions of missed care are more common in survivors reporting PCP-led care builds upon recent findings that survivors are often lost to primary care follow-up and PCP involvement in survivorship care and management of survivorship-related issues remains low beyond the first 5 years post-treatment [2, 3]. Thus, there is much work left to be done to support primary care teams engaging in the care of low-risk cancer survivors longer-term.

We do not know whether patient perceptions of what aspects of care were missed align with services that are clinically indicated. It is possible that patients’ desire for non-recommended services not being met is driving their perceptions of key aspects of their care being missed. However, in this cohort, we did not see associations between patient-reported receipt of non-recommended tumor marker testing or imaging for recurrence and their report of missed care. Both measures of patient-reported receipt of non-recommended testing were associated with perceptions of duplication. This could be due to patients who receive non-recommended testing being more likely to request services (whether clinically indicated or not) from multiple clinicians, thus leading to more duplication in care. Alternatively, it might reflect care from clinicians who tend to order more interventional care in general, which would be consistent with ordering non-recommended tests.

Our findings reiterate that high-quality survivorship care more than 5 years out from treatment cannot be successfully delivered in the absence of sufficient care coordination between primary care and oncology care teams. Given there are notable gaps in coordination among this lower-risk cohort of survivors with favorable-prognosis disease and limited surveillance requirements, there are likely more significant issues with duplication and coordination in patients with higher-risk and/or more advanced disease. Efforts to more accurately measure care coordination during survivorship and characterize care duplication and fragmentation in other groups of survivors are necessary to target interventions to groups of survivors who most need them. Furthermore, there is little explicit reimbursement for coordination of care, which makes it an unfunded mandate for over-burdened clinicians of all specialties. Leveraging scalable interventions that support primary care involvement, deploying system-level strategies that support the implementation of risk-stratified survivorship care [34–36], and employing evidence-based interventions like care navigation, which have been shown to improve care coordination and patient satisfaction [37], are all important next steps to improving longer-term survivorship care delivery. Efforts to increase reimbursement for care coordination, such as new billing codes that reflect longitudinal follow-up and complexity, should also be prioritized [38].

There are potential limitations to this study that warrant comment. First, this study relied on patient-reported measures of duplication and missed care in survivorship care, and thus, these measures may be subject to recall bias. However, we specifically asked about these coordination issues in the past 2 years to minimize the potential for recall bias and have found patient-report to be a reliable measure of care receipt in our past studies in this cohort [39]. Second, we only asked broadly about whether survivors perceived they experienced these coordination issues. Thus, we cannot make conclusions about what services were specifically duplicated and/or missed as they may not reflect their actual utilization. It is possible that duplicated orders did not result in duplicated care (for example, mammogram was ordered by two different clinicians but the patient did not undergo mammography twice) and that patients wanted interventions that were not offered because they are not supported by evidence-based practice guidelines (for example, surveillance CT scans and biomarkers in patients with early-stage breast cancer) [40]. Third, a small percentage of our sample (2%) did not report seeing a primary care or oncologist in the past 2 years; thus, they may have had less opportunity for cross-clinician duplication, potentially resulting in a more conservative estimate. However, the majority of the sample saw both an oncologist and PCP in the past 2 years and duplication could have resulted from other clinical contacts not reported (e.g., phone calls, portal messages), likely minimizing the potential of this bias. Fourth, this study was partially conducted during the COVID19 pandemic, which has been found to influence the receipt of breast cancer survivorship care [41]. However, we asked patients to recall duplication and missed care in the past 2 years, which included a pre-COVID time period, and adjustment for patient-reported impact of the COVID19 pandemic did not meaningfully change our results. Finally, this study was conducted in two geographically distinct areas of the USA; thus, generalizability may be limited.

A notable proportion of longer-term breast cancer survivors reported their breast cancer survivorship care was duplicated and/or key aspects were missed more than 5 years after initial treatment. Women of color were more likely to report poorer care coordination. Improving coordination between oncology and primary care teams could help mitigate existing duplication and fragmentation in survivorship care. Scalable, sustainable strategies and incentives to promote coordination across oncology and primary care teams later into survivorship are needed to promote equitable delivery of high-quality survivorship care.

## Supplementary Information

Below is the link to the electronic supplementary material.

ESM 1 (DOCX 162 KB)

## Data Availability

The iCanCare Follow-up Study dataset will be made available by the study Principal Investigator (Wallner) upon request.

## Declarations

### Disclaimer

The ideas and opinions expressed herein are those of the authors. The State of California, Department of Public Health, the NCI, and the CDC and their Contractors and Subcontractors had no role in the design and conduct of the study; collection, management, analysis, and interpretation of the data; and preparation, review, or approval of the manuscript.

### Prior presentations

Preliminary results presented at Cancer Primary Care Research International Network Conference in Oxford, UK (March 2023). Duplication and Fragmentation in Breast Cancer Survivorship Care across Primary Care and Oncology in the USA.
