# Supplementary material for: Patient-reported duplication and missed breast cancer survivorship care more than 5 years after initial treatment
Source: J Cancer Surviv. Author manuscript; Available in PMC 2026 Sep 24. (PMC13602151; doi:10.1007/s11764-026-02038-w)
Supplement: Supplementary file 1 [file NIHMS2206664-supplement-Supplemental_Table_1.docx]

**Supplemental Table 1: Patient-reported utilization of non-recommended testing and perceptions of duplication and missed survivorship care (N=968)**

|  | **Patient-reported Duplication** | | | | **Patient-reported Missed Care** | | | |
| --- | --- | --- | --- | --- | --- | --- | --- | --- |
| **Patient-reported utilization** | No  N | Weighted % | Yes  N | Weighted % | No  N | Weighted % | Yes  N | Weighted % |
| **CA-125 or CEA tests**  Did not receive test in past 5 years  Received test in past 5 years  *p-values* | 367  272 | 77.6  64.0  *<0.001* | 101  150 | 22.4  36.0 | 324  301 | 68.9  71.4  *0.44* | 144  121 | 31.1  28.6 |
| **Imaging for recurrence (CT, PET, bone scan)**  Did not receive test in past 5 years  Received test in past 5 years  *p-values* | 462  203 | 75.5  63.8  *0.001* | 144  115 | 24.5  36.2 | 435  219 | 71.4  69.6  *0.60* | 171  99 | 28.6  30.4 |
